# Large-scale plasma proteomics reveals bidirectional associations between sleep patterns and inflammatory bowel disease: a prospective cohort study

**DOI:** 10.1186/s12916-025-04539-4

**Published:** 2025-12-05

**Authors:** Kaixing Le, Yaru Liu, Rongpan Bai, Jinghao Sheng, Jie Hu, Jinpiao Zhu, Nick Powell, Yang Bi, Daqing Ma, Zhigang Liu

**Affiliations:** 1 National Clinical Research Center for Child Health, Children’s Hospital, Zhejiang University School of Medicine, Hangzhou, 310052 China; 2 Zhejiang Key Laboratory of Neonatal Diseases, Hangzhou, 310052 China; 3 Institute of Environmental Medicine and Department of General Surgery, Sir Run Run Shaw Hospital, Zhejiang University School of Medicine, Hangzhou, 310058 China; 4 Liangzhu Laboratory, Zhejiang University, Hangzhou, 311121 China; 5 Cancer Center, Zhejiang University, Hangzhou, 310058 China; 6 Department of Anesthesiology, Luoyang Central Hospital Affiliated to Zhengzhou University, Luoyang, Henan 471000 China; 7 Department of Metabolism, Digestion and Reproduction, Faculty of Medicine, Imperial College London, London, SW7 2AZ UK; 8 Division of Anesthetics, Pain Medicine and Intensive Care, Department of Surgery and Cancer, Chelsea and Westminster Hospital, Faculty of Medicine, Imperial College London, London, SW10 9NH UK

**Keywords:** Inflammatory bowel disease, Sleep, Proteomics, Insomnia

## Abstract

**Background:**

Sleep disturbances are common in individuals with inflammatory bowel disease (IBD) and may worsen its progression. This study investigates the bidirectional association between unhealthy sleep and IBD and explores proteomic mechanisms underlying this relationship.

**Methods:**

Data from 381,228 UK Biobank participants were analyzed to calculate adjusted odds ratios (ORs) for prevalent IBD and hazard ratios (HRs) for IBD incidence in relation to sleep patterns. A subset of 40,392 participants underwent plasma proteomic profiling, where differential expression analysis and weighted gene co-expression network analysis (WGCNA) identified key protein modules. A prognostic risk model for IBD was developed using least absolute shrinkage and selection operator (LASSO)-Cox regression.

**Results:**

At baseline, 26.4% of participants exhibited unhealthy sleep, which was significantly associated with higher odds of prevalent IBD (*OR* = 1.250, 95% *CI* 1.165–1.340, *p* < 0.001) and an increased risk of developing IBD (*HR* = 1.237, 95% *CI* 1.136–1.348, *p* < 0.001). Proteomic profiling revealed 182 differentially expressed proteins common to both unhealthy sleep and IBD, with WGCNA identifying modules enriched in pathways related to cell activation, chemotaxis, and amino acid and organic acid metabolism. The proteomic risk model achieved an AUC of 0.81 for predicting 2-year IBD onset. Participants with both unhealthy sleep and high proteomic risk scores had a markedly increased risk of incident IBD (*HR* = 3.370, 95% *CI* 2.300–4.938, *p* < 0.001).

**Conclusions:**

Unhealthy sleep and IBD are bidirectionally linked, with inflammatory and metabolic processes mediating this association. These findings highlight potential biomarkers and therapeutic targets, underscoring the importance of integrating sleep assessments into IBD management strategies.

**Supplementary Information:**

The online version contains supplementary material available at 10.1186/s12916-025-04539-4.

## Background

Inflammatory bowel disease (IBD), a chronic autoimmune condition with two main subtypes, ulcerative colitis (UC), and Crohn’s disease (CD), continues to impose a significant global health burden due to its rising prevalence and long-term complications [1–3]. Characterized by recurrent inflammation of the gastrointestinal tract, IBD is influenced by genetic susceptibility, immune dysregulation, and various environmental factors [4]. Among these contributors, sleep has gained growing attention for its potential role in IBD onset and progression [5].

Sleep disorders are diverse and can be broadly classified into several types, including insomnia (difficulty initiating or maintaining sleep), obstructive sleep apnea (repeated interruptions in breathing during sleep), circadian rhythm sleep–wake disorders (misalignment between internal biological rhythms and external environment), hypersomnia (excessive daytime sleepiness), and movement-related disorders such as restless legs syndrome [6]. These disturbances may influence immune function and inflammatory processes, both of which are central to IBD pathogenesis.

Emerging evidence supports the connection between specific types of sleep disorders and IBD. Insomnia has been linked to elevated levels of systemic inflammation [7, 8]. Obstructive sleep apnea has been associated with increased pro-inflammatory cytokines, including TNF-α and IL-6, which are known to play a critical role in IBD pathophysiology [9, 10]. Circadian rhythm disruptions, such as those experienced by shift workers, may alter gut microbiota composition and intestinal permeability, thereby contributing to mucosal inflammation [11]. Additionally, hypersomnia and excessive fatigue are commonly reported among individuals with IBD, often reflecting poor sleep quality or fragmentation [12].

In some studies, poor sleep quality has been reported in over 70% of individuals with active IBD and over 45% of those with quiescent disease, with a higher prevalence among patients with CD [13, 14]. Epidemiological studies suggest that insufficient or disrupted sleep may not only exacerbate disease activity but also increase the risk of developing IBD. For instance, suboptimal sleep has been associated with increased rates of disease flare, hospitalization, and disease-related complications [15].

Experimental models provide additional support for the biological impact of sleep disturbances on intestinal inflammation. Studies in colitis-induced mice have shown that sleep deprivation aggravates gut inflammation, with histological damage and elevated tissue myeloperoxidase (MPO) activity [16, 17]. Mechanistically, poor sleep has been found to trigger the release of pro-inflammatory cytokines such as TNF-α and IL-6, disrupt circadian regulation of immune responses, and create a pro-inflammatory environment that perpetuates intestinal damage and immune activation [9, 10]. These immune-modulating effects of sleep disturbances suggest a potentially bidirectional and self-perpetuating cycle between sleep dysfunction and IBD activity [18].

While prior studies have established an epidemiological and mechanistic link between sleep disturbances and IBD, the molecular pathways, particularly those reflected in proteomic signatures, remain poorly understood. The UK Biobank, with its extensive plasma proteomic profiles from over 54,000 participants, provides a unique opportunity to explore this relationship at a molecular level [19]. In this study, we aimed to investigate the proteomic signatures associated with various sleep patterns and their relationship with UC and CD. Additionally, we explored potential inflammatory and metabolic protein mediators that may underlie the association between unhealthy sleep and incident IBD. A deeper understanding of these pathways may contribute to early diagnosis, individualized prevention strategies, and novel therapeutic approaches for IBD through the lens of sleep.

## Methods

### Study design and participants

The UK Biobank is a large prospective cohort study that recruited around half a million participants across England, Scotland, and Wales between 2006 and 2010. Health-related data was collected from participants through touchscreen questionnaires, physical measurements, biological samples, and electronic health records.

### Clinical cohort

We established a clinical cohort involving participants with complete sleep pattern records at enrollment to investigate the bidirectional relationship between sleep patterns and IBD (Additional file 1: Fig. S1). Participants meeting the following criteria were excluded: incomplete responses to the sleep questionnaire (*N* = 92,230), extreme sleep durations (< 4 h or > 12 h) (*N* = 917), diagnosis of noninfective gastroenteritis and colitis (*N* = 19,371), missing first occurrence date of IBD (*N* = 2), and inconsistent gender status (*N* = 8521). Disease diagnoses were sourced from hospital inpatient records using the International Classification of Diseases, 10th revision (ICD-10). The classification of IBD was based on the first ICD-10 record of either Crohn’s disease (CD, K50) or ulcerative colitis (UC, K51) as documented in the UK Biobank database. For patients with both diagnoses recorded, we considered the first diagnosis entry in our analysis. Participants diagnosed with K52 (other noninfective gastroenteritis and colitis), which were under the same class as K51 (UC) and K50 (CD), were excluded to avoid bias due to undefined diagnosis and comorbidity. The UK Biobank also summarized the dates of the first occurrences mapped to ICD-10 codes from various sources, including primary care data, hospital inpatient data, death register records, and self-reported medical conditions. Prevalent and incident IBD cases can be distinct by comparing the first occurrence date of UC (K51) and CD (K50) to the enrolled date. After further filtering out participants with sex mismatch and sex chromosome aneuploidy, referring prerequisites of omics studies, data from 381,228 participants were included in the clinical cohort.

### Proteomic cohort

A subpopulation from the UK Biobank underwent plasma proteomic profiling at enrollment as part of the UKB-PPP project [19]. Normalized expression data covering 2923 plasma proteins were released to UK Biobank applicants. After quality control, 408 participants with outlier proteomic values and 12 proteins with over 20% missing values were excluded. The final dataset included 2911 proteins from 40,392 participants, irrespective of IBD status, to investigate proteomic signatures and mediating mechanisms between sleep and IBD (Additional File 1: Fig. S1). Participants without prevalent IBD were randomly divided into training and validation cohorts to establish a predictive model for incident IBD.

### Assessment of sleep behaviors

Seven sleep behaviors were assessed using a touchscreen questionnaire during the enrollment visit, including chronotype, sleep duration, insomnia, daytime sleepiness, snoring, getting up time, and napping during the day. Our study primarily focused on the first five behaviors (excluding getting up time and napping) to assess overall sleep patterns, based on previous literature [20–22]. The content of the questions and the binary scoring of each sleep behavior are described in Additional File 2: Table S1. Four sleep behaviors (chronotype, sleep duration, insomnia, and daytime sleepiness) were consistently associated with both incident and prevalent IBD and were used to create a healthy sleep index score. Each sleep behavior was scored as 1 if the participant met the criteria: an early chronotype (“morning” or “morning-er than evening”), 7–8 h of sleep per day, rarely experiencing insomnia, and no excessive daytime sleepiness. These individual scores were then combined to form a total sleep score. Participants with a total sleep score of 0 or 1 were grouped due to the small number of individuals in these categories.

### Covariate assessment

Given the influence of demographic and lifestyle factors on both IBD and sleep patterns, we obtained information on various covariates, including age (continuous in years), sex (male or female), ethnicity (White or non-White), body mass index (BMI, continuous in kg/m^2^), Townsend deprivation index (TDI, with higher scores indicating higher deprivation), physical activity (calculated as total metabolic equivalent task (MET) hours per week), healthy diet score (with higher scores indicating a healthier diet), alcohol intake (never, ≤ 2 drinks per week, > 2 drinks per week), smoking status (never, previous, current), and polygenic risk scores (PRS) for UC and CD [23, 24]. Sociodemographic factors were collected using touchscreen questionnaires at baseline. Healthy diet scores were estimated by recalling the frequency of consumption of 12 food items over the previous year, using the UK Biobank diet frequency questionnaire scoring approach [25]. PRS for relevant traits were generated based on previously established methods [26].

### Proteomic profiling and metabolic biomarkers

Plasma proteomic profiling was performed using antibody-based Olink Explore 3072 Proximity Extension Assay (PEA) as part of the UKB-PPP. Stringent quality control was applied, and the proteomic data was provided as normalized values. Details of the sample and data processing were described elsewhere [19]. Proteins with more than 20% missing values and participants with proteomic outlier profiles were excluded. For proteins with missing data due to missingness (i.e., data not recorded), we applied mean imputation. For proteins that were below the detection limit, we excluded them from the analysis to prevent bias associated with inappropriate imputation. The final dataset included 2911 proteins.

For sensitivity analyses, proteomic measurements below the assay-specific limit of detection (LOD) were retained rather than excluded. Following a commonly applied strategy in proteomics, these values were imputed with half of the LOD for each protein. Where assay-specific LOD values were unavailable, the lowest non-missing value for that protein was used as a proxy, and missing values were replaced with half of this value. The subsequent differential expression analysis and LASSO–Cox modelling were repeated using this alternative imputation strategy to assess the robustness of the findings.

Metabolic biomarkers were measured using non-fasting baseline EDTA plasma samples analyzed by nuclear magnetic resonance (NMR) spectroscopy (Nightingale Health Ltd., Finland) [27–29]. The normalized concentrations of amino acids and organic acids were included to supplement the analysis of enriched metabolic pathways [30].

### Differential proteins and functional modules

Differentially expressed proteins (DEPs) associated with unhealthy sleep, UC, and CD were identified using the “limma” package in R, with adjustments made for confounders similar to those used in the clinical cohort. The significance threshold was set at a false discovery rate (FDR) of < 0.05. Functional enrichment analysis was conducted using the “clusterProfiler” package after converting protein names to Entrez IDs.

The weighted gene co-expression network analysis (WGCNA) was used to cluster DEPs into functional modules [31]. A soft threshold was selected based on the scale-free topology model fit (*R*^2^ = 0.85), and modules were identified using the blockwiseModules function with a minimum module size of 20. Seven protein modules covering 842 proteins were identified, and functional annotations were summarized based on significant enrichment of KEGG and Gene Ontology Biological Process (GOBP) terms (Additional File 1: Fig. S2). Protein–protein interaction networks were visualized using the “igraph” package, with nodes representing proteins and edges indicating correlations above a specified threshold.

### Mediation analysis

Mediation analysis was conducted to evaluate whether plasma proteins mediated the relationship between sleep and IBD. The proportion mediated by individual protein levels or functional modules was estimated using the “mediation” function in the “bruceR” package, with a nonparametric bootstrap method (1000 draws) to calculate 95% confidence intervals. For time-to-event data, the “CMAverse” package was used to incorporate the proteomic risk score as a mediator.

### LASSO-Cox regression model

Proteins significantly associated with unhealthy sleep (Bonferroni-adjusted *p*-value < 0.05/2911) were selected for least absolute shrinkage and selection operator (LASSO)-Cox regression using the “glmnet” package [32]. The penalty parameter (*λ*) was optimized by tenfold cross-validation, resulting in a value of 7.16E-4 (Additional File 1: Fig. S3). The selected proteins were incorporated into a Cox model for incident IBD, followed by stepwise selection using the Akaike information criterion (AIC). The final protein score was calculated based on coefficients from the Cox model, and predictive performance was assessed using the concordance index (C-index) and area under the curve (AUC) at multiple time points.

### Statistical analysis

Hazard ratios (HR) for prospective analyses and odds ratios (OR) for case–control analyses were reported. Logistic regression was used to estimate ORs for prevalent IBD and unhealthy sleep patterns, while Cox proportional hazards models estimated HRs for incident IBD. Sleep behaviors and IBD subtypes were included as separate outcomes or exposures without mutual adjustments. Missing data for continuous covariates were imputed using sex-specific mean values, and missing categorical covariates were imputed using the most frequent category (Additional File 2: Table S2). Confounders in the adjusted models included age, sex, ethnicity, BMI, smoking status, alcohol intake, TDI, diet score, and physical activity [33, 34]. Stratified analyses and interaction effects across subgroups were also examined. Sensitivity analyses excluded IBD patients diagnosed within 2 years of enrollment and excluded shift workers. All analyses were performed using R software (version 4.3.2).

## Results

### Unhealthy sleep and IBD incidence were bidirectionally associated

**Fig. 1:**
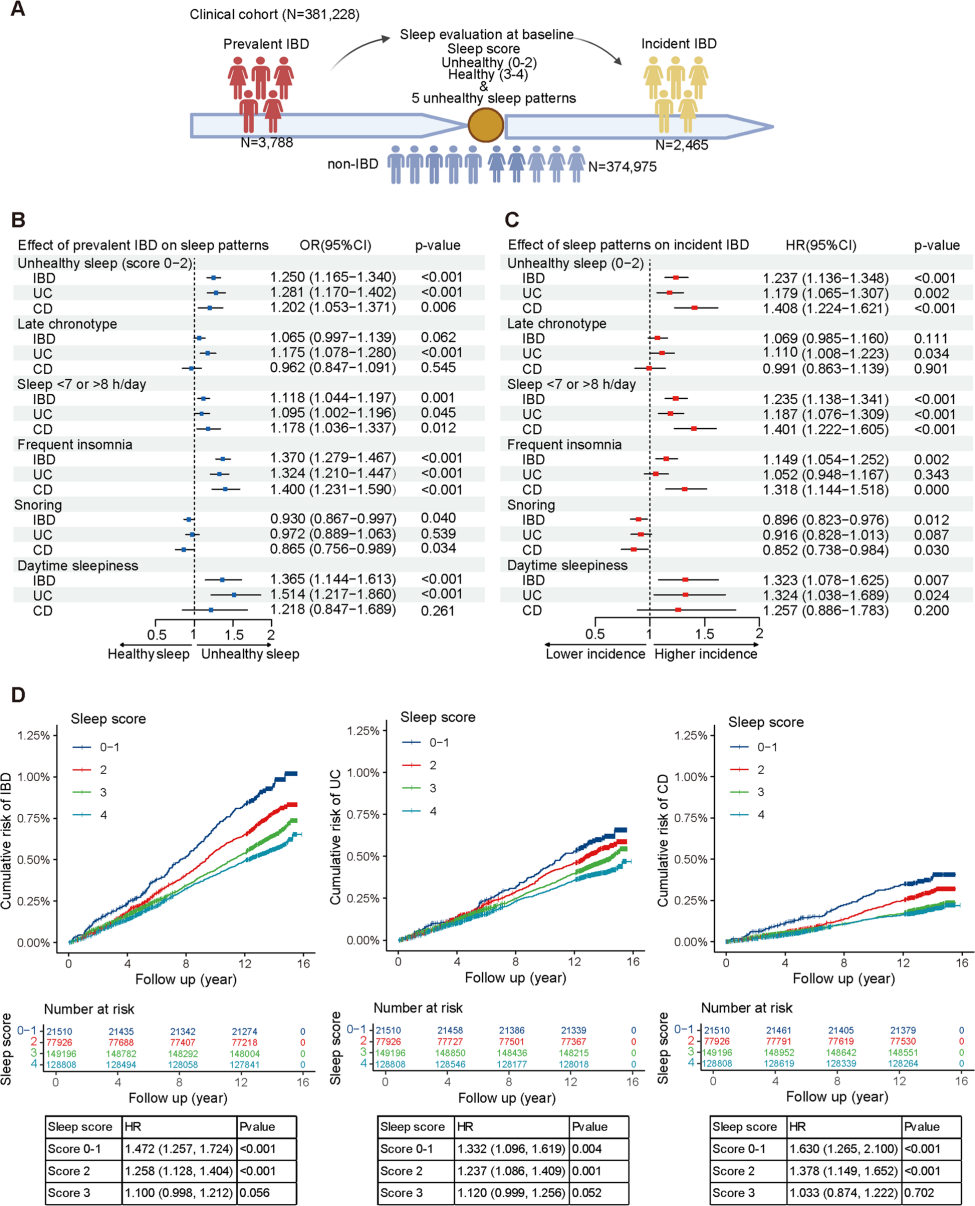
Bidirectional association between sleep and IBD. **A** Study design of the clinical cohort to evaluate the bidirectional relationship between sleep and IBD. Forest plots show associations between baseline sleep patterns and prevalent IBD (**B**) and the risk of incident IBD (**C**). Odds ratios (OR) were estimated using logistic regression, and hazard ratios (HR) were calculated using Cox regression. Both models adjusted for confounders, including age, sex, ethnicity, BMI, smoking status, alcohol intake, Townsend deprivation index (TDI), diet score, and physical activity. **D** Cumulative risk curves illustrate the risk of incident IBD and its subtypes across varying sleep scores. HRs were estimated using multivariable Cox models with the same covariates adjusted

We analyzed data from an ambispective cohort of 381,228 participants from the UK Biobank to investigate the bidirectional association between unhealthy sleep and IBD. Sleep scores were derived from four self-reported baseline sleep behaviors: chronotype, sleep duration, insomnia, and daytime sleepiness, with possible scores ranging from 0 to 4 points. The distribution of sleep scores was as follows: 5.7% scored 0–1, 20.7% scored 2, 39.5% scored 3, and 34.1% scored 4 (Fig. 1A, Table 1). Sleep scores of 0–2 were classified as indicating “unhealthy sleep.” Among the cohort, 3788 participants had prevalent IBD at recruitment, and 2466 developed incident IBD during a median follow-up period of 13.7 years (*IQR*: 13.1–14.4 years), resulting in 5,166,260 person-years of observation (Fig. 1A). Both prevalence and incidence rates of UC and CD were significantly higher in participants with lower sleep scores (Table 1).

**Table 1:** Characteristics of the clinical cohort according to the quintiles of healthy sleep scores

Baseline characteristics	Healthy sleep score				*p*-value
	0–1	2	3	4	
**Number of participants**	21,817	78,805	150,694	129,912	
**Age (years)**	56.6 (7.9)	56.8 (8.0)	56.2 (8.2)	56.4 (8.1)	< 0.0001
**Female (%)**	59.9	57.2	52.7	54.1	< 0.0001
**White (%)**	83.0	84.0	84.3	85.5	< 0.0001
**BMI (kg/m**^**2**^**)**	28.5 (5.5)	27.8 (5.0)	27.3 (4.6)	26.9 (4.4)	< 0.0001
**IBD (%)**					< 0.0001
Prevalent IBD	1.41	1.12	0.99	0.85	
Incident IBD	0.93	0.75	0.63	0.55	
**UC (%)**					< 0.0001
Prevalent UC	1.01	0.76	0.69	0.59	
Incident UC	0.61	0.56	0.48	0.42	
**CD (%)**					< 0.0001
Prevalent CD	0.49	0.42	0.37	0.31	
Incident CD	0.43	0.33	0.24	0.23	
**Polygenic risk score**					
UC	0.16 (0.99)	0.14 (0.99)	0.13 (0.98)	0.12 (0.99)	< 0.0001
CD	− 0.07 (0.91)	− 0.09 (0.90)	− 0.09 (0.90)	− 0.10 (0.90)	0.0002
**TDI**	− 0.8 (3.3)	− 1.2 (3.1)	− 1.5 (3.0)	− 1.7 (2.9)	< 0.0001
**Physical activity (MET — hour/week)**	38.0 (39.2)	39.9 (39.5)	41 (39.4)	43.4 (40.5)	< 0.0001
**Healthy diet score**	3.3 (1.4)	3.3 (1.4)	3.3 (1.4)	3.4 (1.4)	< 0.0001
**Smoking status (%)**					< 0.0001
Never	46.7	50.9	54.1	59.5	
Previous	37.2	36.6	35.4	33.0	
Current	16.1	12.5	10.5	7.5	
**Alcohol intake (%)**					< 0.0001
Never	9.7	8.0	6.9	7.1	
≤ 2 drinks/week	50.3	48.6	46.9	48.5	
> 2 drinks/week	40.0	43.4	46.2	44.4	

A healthy sleep pattern was scored in aspects of four sleep behaviors: chronotype, sleep duration, insomnia, and daytime sleepiness. Each sleep factor was scored 1 if meeting the following criteria: Early chronotype (“morning” or “morning than evening”), sleep 7–8 h per day, reported never/rarely or sometimes insomnia symptoms, and no excessive daytime sleepiness (“never/rarely” or “sometimes”). Continuous variables were presented as mean (standard deviation). *TDI* Townsend deprivation index

Participants with higher sleep scores [3, 4] were more likely to exhibit healthy behaviors, such as a better diet, more physical activity, and other favorable habits (Table 1). To account for potential confounding, multiple demographic and lifestyle factors were adjusted for in the regression models. These adjustments confirmed the bidirectional association between unhealthy sleep and IBD, as well as the specific associations between sleep patterns and IBD subtypes. Participants with prevalent IBD had significantly higher odds of unhealthy sleep (*OR* = 1.250, 95% *CI* 1.165–1.340, *p* < 0.001) (Fig. 1B). In addition, participants reporting unhealthy sleep at baseline had a significantly higher risk of developing IBD (*HR* = 1.237, 95% *CI* 1.136–1.348, *p* < 0.001) (Fig. 1C). These findings were further confirmed by two sensitivity analyses: one excluding participants diagnosed with IBD within 2 years of recruitment and another excluding shift workers (Additional File 2: Table S3). No significant interactions were found across subgroups based on covariates (all p for interaction > 0.05, Additional File 1: Fig. S4). In cumulative risk analyses, the risk of incident IBD progressively increased with deteriorating sleep scores. Participants with sleep scores of 0–1 had the highest cumulative risk of IBD (*HR* = 1.472, 95% *CI* 1.257–1.724, *p* < 0.001), with elevated risks observed for both UC (*HR* = 1.332, 95% *CI* 1.096–1.619, *p* = 0.004) and CD (*HR* = 1.630, 95% *CI* 1.265–2.100, *p* < 0.001) (Fig. 1D). Specific poor-quality sleep patterns, such as frequent insomnia, showed a greater association with prevalent IBD, while shortened or prolonged sleep duration was more significantly associated with incident IBD (Fig. 1B, C). Chronotype and daytime sleepiness were significantly associated with UC but not CD, whereas sleep duration and insomnia showed stronger associations with CD than with UC (Fig. 1C).

### Common differentially expressed proteins and pathways between unhealthy sleep and IBD revealed inflammatory perturbations

**Fig. 2:**
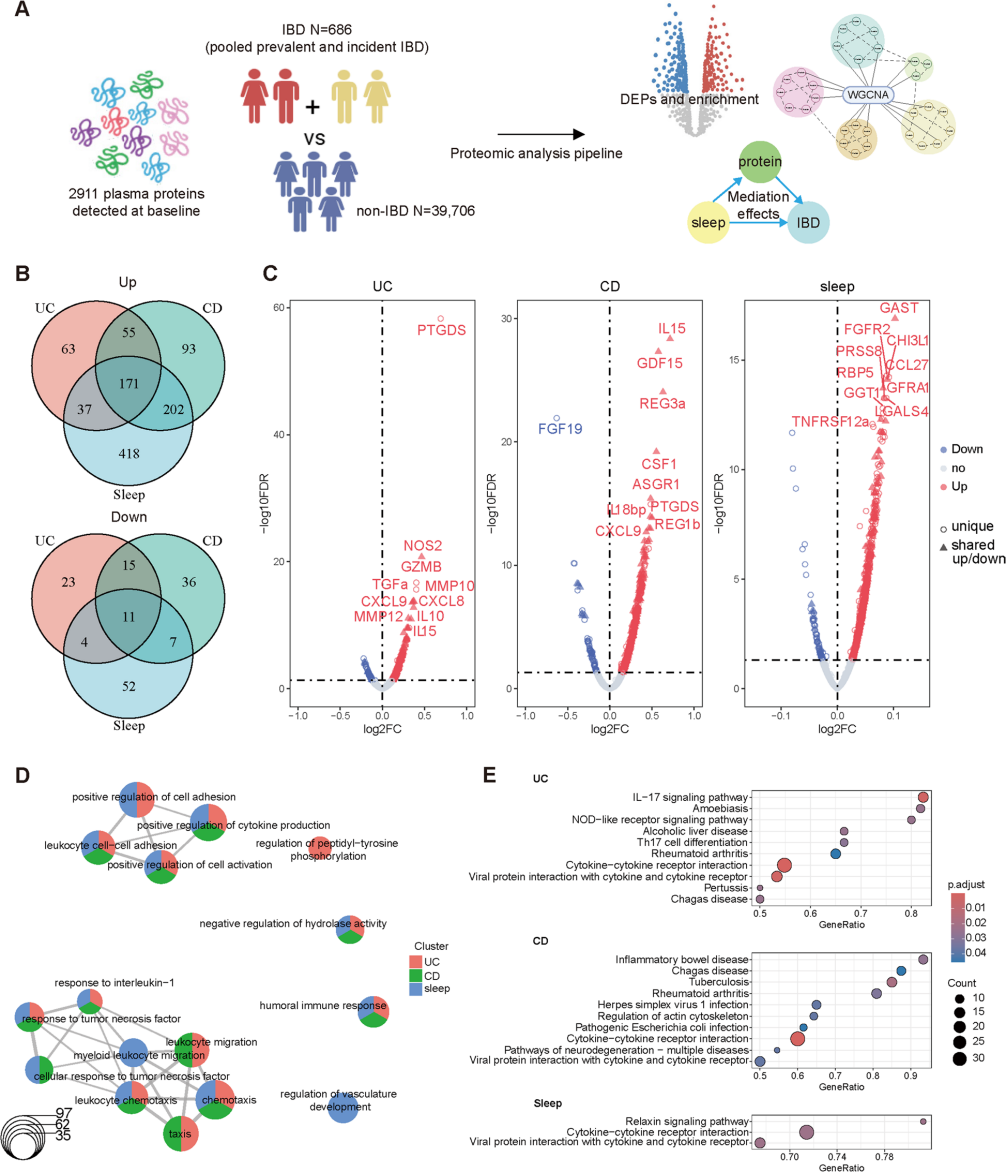
Identification of common DEPs and pathways between sleep and IBD. **A** Overview of the proteomic cohort design and analysis pipeline. **B** Venn diagram showing 171 commonly upregulated and 11 commonly downregulated DEPs across UC, CD, and unhealthy sleep datasets. **C** Volcano plots highlight significant DEPs (*FDR* < 0.05) for UC, CD, and unhealthy sleep. Dots within triangles indicate overlapping proteins across all three phenotypes, while circles represent unique proteins. Gene Ontology Biological Process (GOBP) and KEGG GSEA enrichment analyses were performed for each dataset. **D** Enrichment map clustering GOBP terms associated with shared protein sets, with phenotype-specific terms represented as color-coded nodes. **E** KEGG GSEA enrichment results were visualized as dot plots for each phenotype

A subset of participants (*N* = 40,392) with plasma proteomics data formed the proteomic cohort to investigate the mechanisms underlying the sleep-IBD association (Fig. 2A). A total of 182 common differentially expressed proteins (DEPs), including 171 upregulated and 11 downregulated proteins, suggested a shared proteomic signature between sleep and IBD (Fig. 2B). Compared to UC, CD shared more proteins with unhealthy sleep, including 202 elevated and 7 lowered proteins. Overlapping DEPs between unhealthy sleep, UC, and CD were highlighted in separate volcano plots (Fig. 2C). Many of the shared proteins in CD, UC, and unhealthy sleep were involved in regulating inflammation and maintaining intestinal homeostasis (Additional File 2: Table S4). For instance, upregulated C-X-C motif chemokine ligand 8 (CXCL8), cytokine interleukin-15 (IL-15), and chitinase-3-like protein-1 (CHI3L1) were common pro-inflammatory markers. Interestingly, gastrin (GAST), a hormone involved in hydrochloric acid secretion, was among the top upregulated proteins in unhealthy sleep and was also linked to IBD (Fig. 2C). Functional enrichment analysis of these DEPs was conducted using Gene Ontology Biological Process (GOBP) and gene set enrichment analysis (GSEA) of Kyoto Encyclopedia of Genes and Genomes (KEGG) (Fig. 2D and E). The enriched pathways related to inflammation included positive regulation of cytokine production, cell activation, leukocyte adhesion, and chemotaxis, highlighting the immune activation underlying unhealthy sleep and IBD (Fig. 2D). Consistent with these findings, separate pathway analysis further revealed that inflammatory perturbations underlie both unhealthy sleep and IBD, with significant enrichment in pathways such as the interleukin-17 (IL-17) signaling pathway and Th17 cell differentiation (Fig. 2E).

### Functional proteomic modules highlighted distinct mediations of sleep in UC and CD

**Fig. 3:**
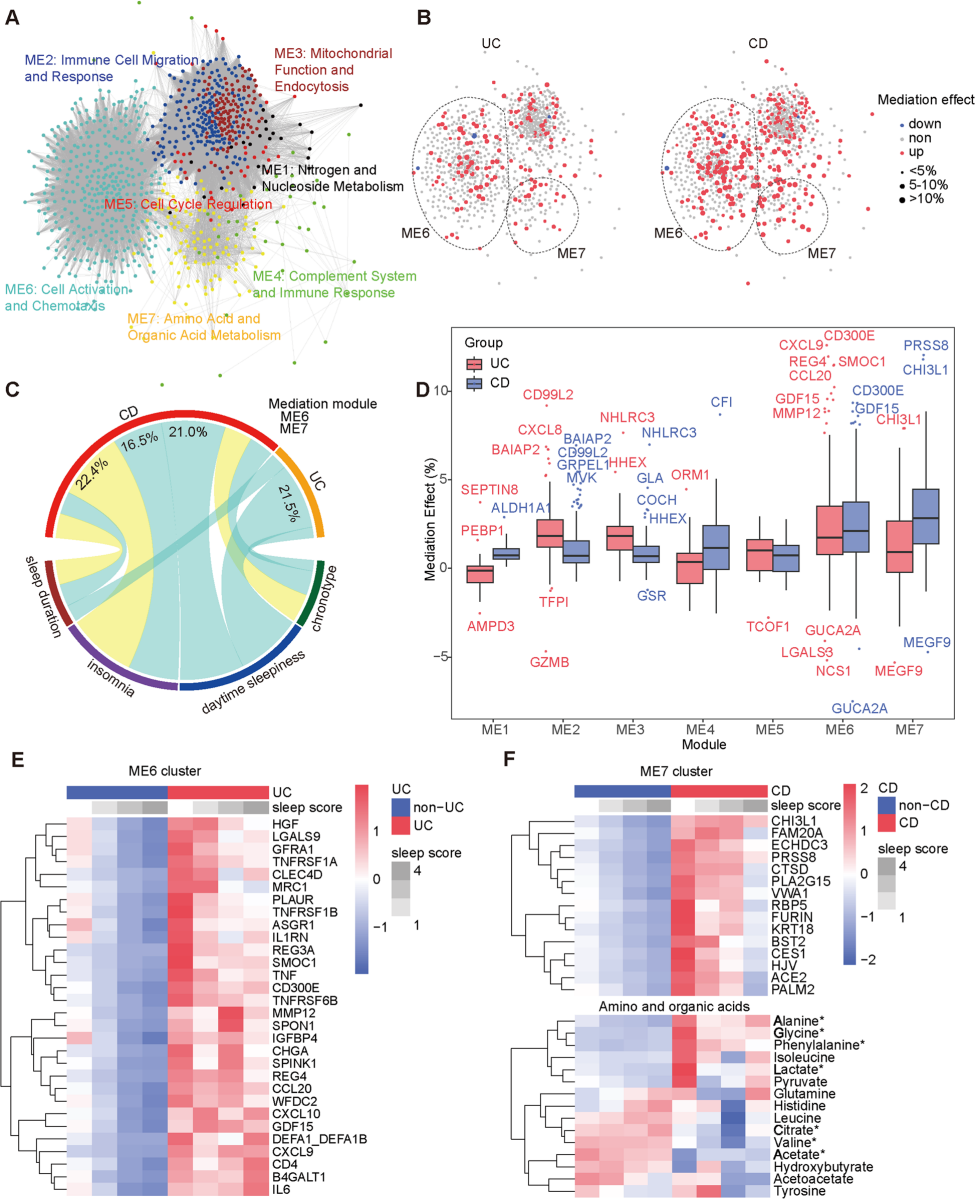
Functional modules mediating the crosstalk between sleep and IBD. DEPs identified from UC, CD, or sleep datasets were clustered using WGCNA. **A** WGCNA identified seven functional modules (ME1–ME7) enriched with DEPs, with each network node representing one protein, color-coded by functional module. **B** Overlay of proteins with significant mediation effects between sleep and UC (left) or CD (right) on the network nodes. **C** Chord diagram illustrating the mediation effects of modules ME6 and ME7 on different sleep traits and IBD, with arc widths representing relative mediation strength. **D** Boxplot comparing the mediation effects of proteins between UC and CD across clusters. **E** Heatmap of the top 30 significant proteins in ME6 showing scaled average levels across different sleep scores and UC status. **F** Heatmap of the top 15 significant proteins in ME7 and all detected amino and organic acids showing scaled averages across different sleep scores and CD status

To better characterize proteomic hallmarks correlating with clinical phenotypes, we performed WGCNA on DEPs from UC, CD, and unhealthy sleep, identifying seven module eigengenes (ME1–7) representing independent functional entities (Fig. 3A; Additional File 2: Table S4). Inflammatory processes predominated in ME2 (immune cell migration and response), ME4 (complement system and immune response), and ME6 (cell activation and chemotaxis). Metabolic processes were observed in ME1 (nitrogen and nucleoside metabolism) and ME7 (amino acid and organic acid metabolism). Overlaying proteins with significant mediation effects between sleep and UC or CD illustrated stronger mediation effects and more mediators for CD, indicating more pronounced proteomic mediation of unhealthy sleep in CD (Fig. 3B). Most of these proteins showed positive mediation effects. Compared to UC, ME7 had a higher mediation effect on CD (Fig. 3D). CXCL9, within ME6, significantly mediated the association between unhealthy sleep and UC (mediation effect: 11.95%, *p* < 0.001) (Fig. 3D; Additional File 2: Table S5). Similarly, CHI3L1 in ME7 had a significant mediation effect (11.79%, *p* < 0.001) on the association between unhealthy sleep and CD (Fig. 3D; Additional File 2: Table S5). Further exploration of the mediation effects of sleep patterns on UC and CD through functional proteomic modules revealed that only ME6 and ME7 had significant mediation effects. ME6 mediated the association between excessive daytime sleepiness and both UC and CD (mediation effect for UC: 21.5%, *p* = 0.015; for CD: 21.0%, *p* = 0.007) (Fig. 3C; Additional File 2: Table S6). Insomnia was significantly associated with CD through ME6 and ME7, with mediation effects of 16.5% (*p* = 0.024) and 22.4% (*p* = 0.002), respectively. Sleep duration was correlated with both IBD subtypes through different modules: ME6 (mediation effect: 7.3%, *p* = 0.002) and ME7 (mediation effect: 8.0%, *p* = 0.002) for CD and ME6 (mediation effect: 5.4%, *p* = 0.019) for UC. This suggests that poor sleep was linked to UC primarily through inflammation, while CD was associated with both inflammatory activation and amino acid/organic acid metabolism. We mapped the correlations of proteins in the ME6 and ME7 clusters with UC and CD, respectively (Fig. 3E, F). Proteins in ME6 showed elevated levels in UC patients compared to non-UC participants, with levels decreasing as sleep scores improved, particularly for pro-inflammatory cytokines and receptors such as TNF, TNFRSF1A, TNFRSF1B, TNFRSF6B, and CLEC4D (Fig. 3E). In ME7, proteins were similarly increased in CD patients compared to those without CD and showed decreasing trends with higher sleep scores (Fig. 3F). Analysis of amino and organic acid metabolites showed that levels of alanine, glycine, phenylalanine, and lactate were significantly elevated in CD, while citrate, valine, and acetate were decreased. Levels of these metabolites also decreased with improving sleep scores. These results indicate that inflammatory mediators were involved in both UC and CD, while metabolic mediators were primarily involved in CD.

### Sleep-related proteomics demonstrated strong predictive value for IBD incidence

**Fig. 4:**
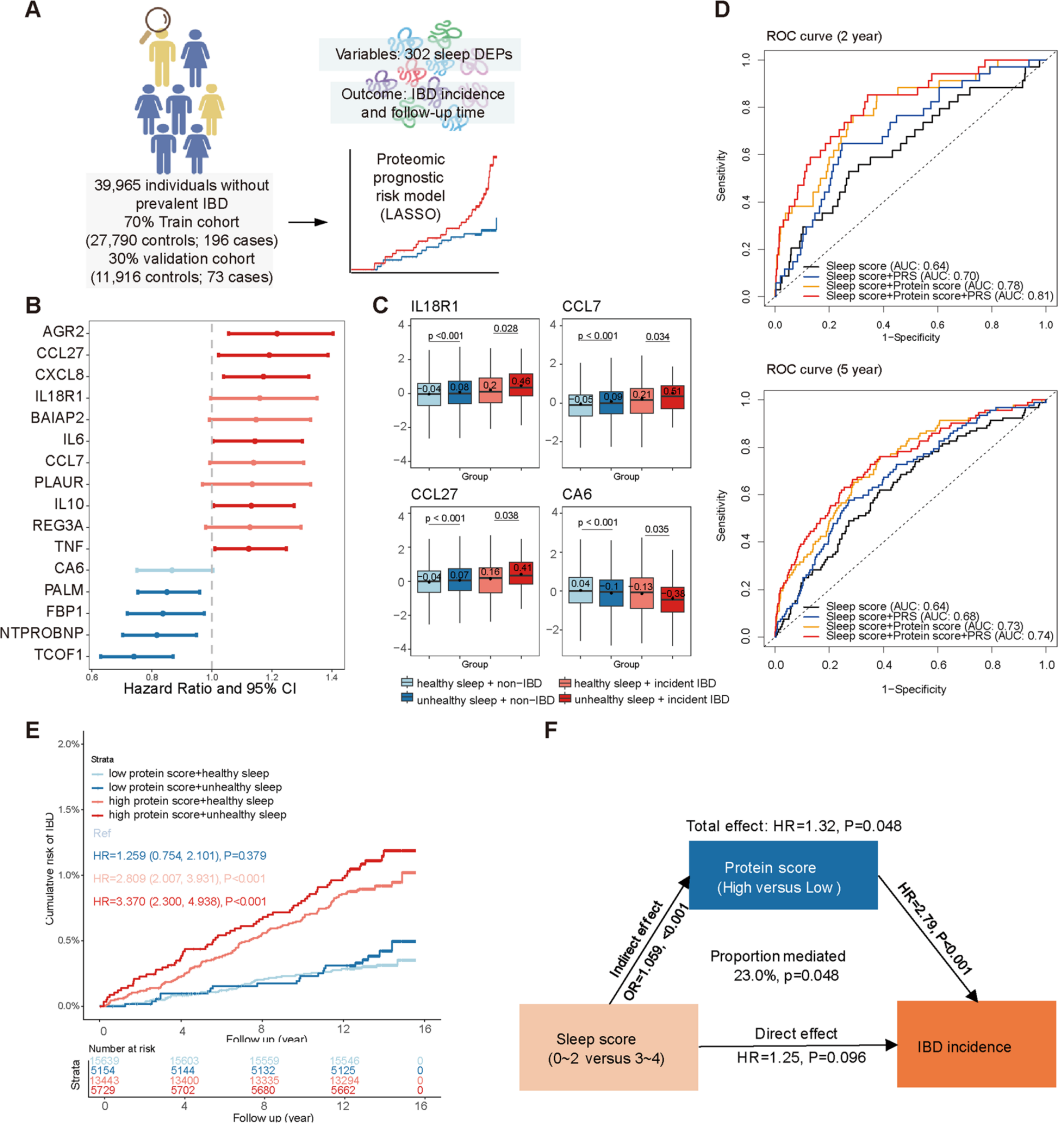
Sleep-related plasma proteins predicting the risk of IBD incidence. **A** Workflow showing the integration of clinical and proteomic datasets to develop a sleep-related prediction model for incident IBD. A total of 302 sleep-related DEPs (Bonferroni-adjusted significance) were included in a Lasso-Cox model. **B** Coefficient plot showing associations between proteins contributing to the protein score and incident IBD. HRs per 1 SD increase in protein levels were calculated using an adjusted Cox model. **C** Comparison of protein levels between poor and healthy sleep groups, stratified by IBD status. Dots represent mean values, and boxplots show the distribution, including mean and quantiles, with significance assessed using a one-tailed *t*-test. **D** ROC curves comparing 2- and 5-year prediction scores across models using sleep scores (demographics and lifestyle covariates), protein score, and PRS individually or combined. **E** Cumulative risk curves for incident IBD in the cohort stratified by proteomic risk and sleep health, with HRs estimated using adjusted Cox models. **F** Mediation analysis showing the protein score’s effect on the relationship between unhealthy sleep and incident IBD, estimated using the Cox model

To predict IBD risk related to unhealthy sleep, we established a prognostic risk model through LASSO-Cox regression analysis. Participants in the proteomic cohort without prevalent IBD (*N* = 39,965) were randomly split into training (70%) and validation (30%) cohorts. Among 302 DEPs significantly associated with unhealthy sleep (Bonferroni-adjusted *p*-value < 0.05), 16 proteins were selected for the final model (Fig. 4A). Of these, 11 proteins were associated with an increased risk of IBD, while 5 indicated a protective effect (Fig. 4B). Elevated levels of interleukin-18 receptor 1 (IL18R1), chemokine C–C motif ligand 7 (CCL7), CCL27, and carbonic anhydrase 6 (CA6) were seen in individuals with unhealthy sleep, regardless of IBD status (Fig. 4C). The protein score, derived from these 16 proteins, demonstrated an AUC of 0.85 for predicting 2-year onset of IBD in the training cohort and an AUC of 0.68 in the validation cohort, outperforming predictions for long-term risk (5–10 years) (Additional File 1: Fig. S5). When combined with sleep scores and IBD polygenic risk scores, the model’s performance improved, achieving AUCs of 0.81 and 0.74 for 2- and 5-year onset predictions, respectively (Fig. 4D). Participants were then stratified into low- and high-protein score groups. Among those with unhealthy sleep, participants in the high-protein score group had a significantly elevated risk of incident IBD (*HR* = 3.370, 95% *CI* 2.300–4.938, *p* < 0.001) (Fig. 4E). Mediation analysis showed that high protein scores accounted for 23.0% of the effect of unhealthy sleep on incident IBD (*p* = 0.048), suggesting that sleep-related proteins played a more significant role than unhealthy sleep itself (Fig. 4F).

Sensitivity analyses imputing below-detection values with half of the LOD yielded results that were highly consistent with the main analyses. The majority of differentially expressed proteins overlapped with the primary dataset (> 90%), and the functional modules identified by WGCNA remained unchanged. Similarly, the prognostic proteomic model retained good performance (*AUC* = 0.82 for 2-year IBD onset, Additional File 1: Fig. S6), with negligible differences from the primary analysis.

## Discussion

This study elucidates the relationship between sleep patterns and IBD incidence based on detailed clinical and proteomic datasets. By analyzing data from an ambispective clinical cohort of 381,228 participants from the UK Biobank, we found that poor sleep is bidirectionally associated with IBD. Further analysis of 2911 baseline plasma proteins from 40,392 participants revealed common differentially expressed proteins and pathways, indicating inflammatory perturbations underlying unhealthy sleep and IBD. By clustering these proteins into functional modules, we discovered that inflammation and protein metabolism mediate the impact of unhealthy sleep on UC and CD. Finally, our proteomic LASSO-Cox model demonstrated the predictive value of unhealthy sleep-related proteins in IBD incidence.

Sleep is a homeostatic physiological process that plays a crucial role in regulating immune function. Previous studies have shown that poor sleep is linked to several immune-mediated diseases, including multiple sclerosis, rheumatoid arthritis, systemic lupus erythematosus, type 1 diabetes, and IBD [35–39]. However, the effects of specific dimensions of sleep, such as chronotype and sleep efficiency, on these autoimmune diseases and their subtypes require further investigation. In this study, we assessed sleep quality using four indexes: chronotype, sleep duration, insomnia, and daytime sleepiness [21]. Unhealthy sleep was defined as a sleep score below 3 points. We found that shortened or prolonged sleep duration and excessive daytime sleepiness were particularly associated with a higher risk of incident IBD, consistent with previous studies [39, 40]. Shortened sleep duration and excessive daytime sleepiness are both indicative of poor sleep quality at night [41]. Sleep deprivation has been shown to activate several pro-inflammatory signaling pathways involving nuclear factor-κB (NF-κB), activator protein-1 (AP-1), signal transducer and activator of transcription (STAT) family proteins, and cytokines such as IL-6 and TNF-α [10, 42, 43]. Similarly, sleep deprivation in mice resulted in severe inflammation and even death due to a cytokine-storm-like syndrome involving increased prostaglandin D2 (PGD2) and the accumulation of circulatory neutrophils [44]. However, the effects of prolonged sleep duration on the immune system and health outcomes remain less well understood.

IBD is characterized by chronic inflammation of the gastrointestinal tract, with pro-inflammatory biomarkers such as C-reactive protein (CRP), fecal calprotectin, and TNF-α elevated during the preclinical and clinical stages [1, 45, 46]. In our study, the plasma proteomics of participants with unhealthy sleep and IBD showed enrichment in pathways related to cytokine production, cell activation, leukocyte adhesion, and chemotaxis, indicating immune activation in both conditions. Notably, the IL-17 signaling pathway was highly enriched in UC patients. Previous studies of colonic mucosa samples have also identified elevated IL-17 expression in active UC and CD patients [47]. Our findings suggest that inflammation mediates the association between sleep disturbances and incident IBD.

Our analysis further revealed differences between UC and CD in terms of proteomic mediators of unhealthy sleep. For UC, inflammation-related modules predominated, including cell activation and chemotaxis. TNF receptor family members, such as TNFRSF1A, TNFRSF1B, and TNFRSF6B, showed decreasing trends as sleep scores improved. TNFRSF1A and TNFRSF1B are both receptors for TNF-α, which play a key role in the inflammatory processes of IBD [48]. TNF-α binding to TNFRSF1A activates intracellular pathways related to apoptosis or survival responses, while TNFRSF1B primarily activates cell survival and proliferation pathways [49]. In contrast to UC, proteomic mediators of the relationship between unhealthy sleep and CD involved both inflammatory and metabolic pathways. For example, amino acids such as phenylalanine were elevated in the plasma of CD patients, consistent with previous findings [50]. Phenylalanine, an aromatic amino acid, has been shown to promote inflammation by triggering alveolar macrophage pyroptosis and cytokine release, though its role in IBD and sleep disturbances remains unclear [51]. Short-chain fatty acids (SCFAs), such as acetate, were decreased in CD patients, suggesting gut dysbiosis and impaired intestinal homeostasis [52, 53].

Our work also identified predictive biomarkers of incident IBD based on plasma proteomics. The LASSO-Cox model proposed in this study performed well in both training and validation cohorts, demonstrating a higher cumulative risk for IBD in individuals with poor sleep quality. This model may serve as a preclinical diagnostic tool for identifying individuals at risk of IBD, providing opportunities for early intervention. Importantly, the model also sheds light on potential mechanisms linking poor sleep to IBD. For instance, pro-inflammatory cytokines (IL-6) and receptors (IL18R1) were elevated in response to unhealthy sleep. Previous studies have shown upregulation of IL-18 and IL18R1 in the brains of sleep-deprived mice, indicating a systemic response to sleep disturbances [54]. IL-18 is primarily produced by intestinal epithelial cells, where it can disrupt gut barrier integrity upon activation by inflammatory stimuli [55]. The role of chemokines, such as CXCL8, CCL7, and CCL27, as mediators of IBD risk was also highlighted by our prognostic model. These chemokines are known to recruit immune cells to inflamed tissues, contributing to tissue destruction in IBD [56–58]. Additionally, alterations in circadian rhythms, which regulate digestive enzymes and gastrointestinal homeostasis, may contribute to the development of IBD. For example, anterior gradient 2 (AGR2), a protein linked to intestinal inflammation and epithelial stress, was among the proteins with the highest hazard ratios in our model [59]. Several of the identified proteins, including IL-6, TNFRSF1A, and ANGPTL4, are involved in pathways targeted by existing or investigational anti-inflammatory and metabolic therapies. These findings raise the possibility that sleep improvement strategies could modulate systemic inflammatory mediators and act synergistically with current IBD treatments. Future studies are warranted to evaluate whether targeted modulation of these pathways could provide therapeutic benefit in patients with disturbed sleep and IBD.

Our study has several limitations. First, sleep behaviors were assessed at baseline, and changes over time during follow-up were not captured. Incorporating longitudinal sleep assessments could provide deeper insights into the trajectory of sleep disturbances and their impact on IBD over time. Second, the proteomic cohort was designed as a case–control study due to the relatively small sample size, and we did not differentiate between prevalent and incident IBD in the proteomic analysis. A separate prospective cohort would help clarify the pathways involved in sleep-related IBD incidence. Third, our classification of IBD was based on the first ICD-10 diagnosis of either CD or UC, and we did not apply a two-diagnosis criterion, which is commonly used to minimize false positives. This approach could potentially lead to misclassification in patients with both diagnoses recorded, which may affect the accuracy of the findings. Fourth, our findings have not been externally validated, which may limit their generalizability. Fifth, when there are many more controls than cases in a prediction model setting, a high AUC could be misleading, as the model might classify all individuals as controls and still achieve a high score. We have evaluated precision, recall, and balanced accuracy, but we acknowledge that class imbalance may still affect the evaluation of model performance. Sixth, we excluded participants with sleep durations of < 4 h or > 12 h, aiming at focusing on the more typical sleep duration range (6–9 h) to avoid including other health issues rather than typical sleep patterns. However, these individuals could represent a group with severe sleep disorders, such as insomnia or sleep deprivation, and excluding them may overlook important aspects of the sleep-IBD relationship. Seventh, our cohort includes a broader general population rather than being limited to individuals with active IBD, which may account for the lower prevalence of unhealthy sleep observed in our study compared to those focused exclusively on IBD patients. Lastly, other factors affecting IBD and sleep, such as psychiatric disorders and gut microbiota, were not assessed, highlighting the need for more comprehensive future studies [60].

## Conclusions

Our study demonstrates that unhealthy sleep is bidirectionally associated with IBD, with inflammatory and metabolic disturbances mediating the relationship. The proteomic model we developed shows promise as a preclinical diagnostic tool for IBD, though further validation is needed to confirm its effectiveness.

## Supplementary Information

Additional file 1. Fig. S1. Flowchart outlining the inclusion process for UK Biobank participants in this study. Fig. S2. Identification of co-expression modules using WGCNA. (A) The dendrogram illustrates protein-level dissimilarity and identifies seven co-expression modules. (B) A heatmap depicts eigengene adjacency among the modules, highlighting their inter-module relationships. (C–D) The soft-thresholding power was set to 3, determined by evaluating the scale-free topology index (C) and mean connectivity (D). Fig. S3. LASSO–Cox regression coefficient selection and variable screening. (A) The lower axis denotes the λ values, and the upper axis indicates the corresponding number of variables retained in the LASSO–Cox model. The λ with the minimum cross-validated error is selected. (B) Ten-fold cross-validation is used to determine the optimal tuning parameter in the LASSO–Cox regression model. (C) The bar plot presents the regression coefficients of the 21 proteins retained at the selected λ. (D) The histogram illustrates the distribution of protein scores, with a threshold defined at zero. Fig. S4. Stratified analysis for the association between sleep and IBD. Stratified analysis was performed according to subgroups of demographics and lifestyle behaviors. The logistic model for OR estimation and the Cox model for HR estimation were adjusted by the same confounders as the main analyses. P.int indicated the significance of differences across subgroups. Fig. S5. Performance assessment of protein score-based predictive models. (A) Receiver operating characteristic curves illustrated the prediction accuracy of the protein score employing 16 proteins in the train and test cohorts. AUC is calculated for different follow-up times separately. (B) Cumulative risk curves compared the incident IBD risk between high protein score and low protein score groups in the training and test cohorts, respectively. HR was calculated by the univariable Cox model. Fig. S6. Receiver operating characteristic curve of the 2-year IBD prediction model incorporating combined sleep scores (with demographic and lifestyle covariates), proteomic score, and polygenic risk score (PRS). Proteomic data included all measured proteins, including those with values below the detection limit (except those of poor assay quality).

Additional file 2. Table S1. UK Biobank touchscreen questionnaire of sleep behaviors and the criteria of the healthy sleep score in this study. Table S2. Missing numbers and rates of variables of the clinical cohort. Table S3. Sensitivity analyses for association between sleep and IBD with different exclusion criteria. Table S4. Differential expression analysis of plasma proteins in the unhealthy sleep and diagnosed UC and CD. Table S5. The 7 functional modules identified by WGCNA and mediation effects of proteins between sleep and IBD. Table S6. Mediation effects of WGCNA modules in the association between sleep patterns and IBD.

## Data Availability

The UK Biobank data are available on application at https:// [www.ukbiobank.ac.uk](http:/www.ukbiobank.ac.uk) /.

## Abbreviations

IBD: Inflammatory bowel disease
UC: Ulcerative colitis
CD: Crohn’s disease
MPO: Myeloperoxidase
TNF-α: Tumor necrosis factor-alpha
IL-6: Interleukin-6
ICD-10: International Classification of Diseases, 10th revision
UKB-PPP: UK Biobank plasma proteomics project
BMI: Body mass index
MET: Metabolic equivalent task
NMR: Nuclear magnetic resonance
DEPs: Differentially expressed proteins
PPI: Protein–protein interaction
MEs: Module eigengenes
LASSO: Least absolute shrinkage and selection operator
AIC: Akaike information criterion
PRS: Polygenic risk score
HR: Hazard ratios
OR: Odds ratios
TDI: Townsend deprivation index
CI: Confidence interval
CXCL8: C-X-C motif chemokine ligand 8
IL-15: Interleukin-15
CHI3L1: Chitinase-3 like-protein-1
GAST: Gastrin
GOBP: Gene Ontology Biological Process
GSEA: Gene set enrichment analysis
KEGG: Kyoto Encyclopedia of Genes and Genomes
IL-17: Interleukin-17
Th17: T helper 17
WGCNA: Weighted correlation network analysis
CLEC4D: C-type lectin domain family 4 member D
IL18R1: Interleukin 18 receptor 1
CCL7: Chemokine C–C motif ligand 7
CCL27: Chemokine C–C motif ligand 27
CA6: Carbonic anhydrase 6
AUC: The area under the curve
IL-8: Interleukin-8
NF-κB: Nuclear factor-κB
AP-1: Activator protein-1
STAT: Signal transducer and activator of transcription
PGD2: Prostaglandin D2
CRP: C-reactive protein
TRADD: TNF receptor-associated death domain protein
SCFA: Short-chain fatty acids
IL-18: Interleukin-18
LPS: Lipopolysaccharide
AGR2: Anterior gradient 2

## References

[1] Plevris N, Lees CW. Disease monitoring in inflammatory bowel disease: evolving principles and possibilities. Gastroenterology. 2022;162(5):1456-75.e1. 35101422 10.1053/j.gastro.2022.01.024

[2] Agrawal M, Allin KH, Petralia F, Colombel JF, Jess T. Multiomics to elucidate inflammatory bowel disease risk factors and pathways. Nat Rev Gastroenterol Hepatol. 2022;19(6):399–409. 35301463 10.1038/s41575-022-00593-y PMC9214275

[3] Ng SC, Mak JWY, Pal P, Banerjee R. Optimising management strategies of inflammatory bowel disease in resource-limited settings in Asia. Lancet Gastroenterol Hepatol. 2020;5(12):1089–100. 33181088 10.1016/S2468-1253(20)30298-3

[4] Singh N, Bernstein CN. Environmental risk factors for inflammatory bowel disease. United Eur Gastroenterol J. 2022;10(10):1047–53. 10.1002/ueg2.12319 PMC9752273 36262056

[5] Qazi T, Farraye FA. Sleep and inflammatory bowel disease: an important bi-directional relationship. Inflamm Bowel Dis. 2019;25(5):843–52. 30388243 10.1093/ibd/izy334

[6] Thorpy MJ. Classification of sleep disorders. Neurotherapeutics. 2012;9(4):687–701. 22976557 10.1007/s13311-012-0145-6 PMC3480567

[7] Sookoian S, Gemma C, Fernández Gianotti T, Burgueño A, Alvarez A, González CD, et al. Effects of rotating shift work on biomarkers of metabolic syndrome and inflammation. J Intern Med. 2007;261(3):285–92. 17305651 10.1111/j.1365-2796.2007.01766.x

[8] Kinnucan JA, Rubin DT, Ali T. Sleep and inflammatory bowel disease: exploring the relationship between sleep disturbances and inflammation. Gastroenterol Hepatol (N Y). 2013;9(11):718–27. 24764789 PMC3995194

[9] Axelsson J, Rehman JU, Akerstedt T, Ekman R, Miller GE, Höglund CO, et al. Effects of sustained sleep restriction on mitogen-stimulated cytokines, chemokines and T helper 1/ T helper 2 balance in humans. PLoS One. 2013;8(12):e82291. 24349251 10.1371/journal.pone.0082291 PMC3859577

[10] Irwin MR, Wang M, Campomayor CO, Collado-Hidalgo A, Cole S. Sleep deprivation and activation of morning levels of cellular and genomic markers of inflammation. Arch Intern Med. 2006;166(16):1756–62. 16983055 10.1001/archinte.166.16.1756

[11] Niu Y, Heddes M, Altaha B, Birkner M, Kleigrewe K, Meng C, et al. Targeting the intestinal circadian clock by meal timing ameliorates gastrointestinal inflammation. Cell Mol Immunol. 2024;21(8):842–55. 38918576 10.1038/s41423-024-01189-z PMC11291886

[12] Salwen-Deremer JK, Reid MJ, Westvold SJ, Siegel CA, Smith MT. People with IBD evidence more microarousals during sleep architecture assessments. BMJ Open Gastroenterol. 2023. 10.1136/bmjgast-2023-001249. 38154825 10.1136/bmjgast-2023-001249 PMC10759128

[13] McGing JJ, Radford SJ, Francis ST, Serres S, Greenhaff PL, Moran GW. Review article: the aetiology of fatigue in inflammatory bowel disease and potential therapeutic management strategies. Aliment Pharmacol Ther. 2021;54(4):368–87. 34228817 10.1111/apt.16465

[14] Graff LA, Vincent N, Walker JR, Clara I, Carr R, Ediger J, et al. A population-based study of fatigue and sleep difficulties in inflammatory bowel disease. Inflamm Bowel Dis. 2011;17(9):1882–9. 21830266 10.1002/ibd.21580

[15] Barnes A, Spizzo P, Bampton P, Andrews JM, Fraser RJ, Mukherjee S, et al. Examining the influence of inflammatory bowel disease medications on sleep quality. JGH Open. 2023;7(3):190–6. 36968569 10.1002/jgh3.12871 PMC10037038

[16] Preuss F, Tang Y, Laposky AD, Arble D, Keshavarzian A, Turek FW. Adverse effects of chronic circadian desynchronization in animals in a “challenging” environment. Am J Physiol Regul Integr Comp Physiol. 2008;295(6):R2034-40. 18843092 10.1152/ajpregu.00118.2008 PMC2685296

[17] Tang Y, Preuss F, Turek FW, Jakate S, Keshavarzian A. Sleep deprivation worsens inflammation and delays recovery in a mouse model of colitis. Sleep Med. 2009;10(6):597–603. 19403332 10.1016/j.sleep.2008.12.009 PMC3509796

[18] Orr WC, Fass R, Sundaram SS, Scheimann AO. The effect of sleep on gastrointestinal functioning in common digestive diseases. Lancet Gastroenterol Hepatol. 2020;5(6):616–24. 32416862 10.1016/S2468-1253(19)30412-1

[19] Sun BB, Chiou J, Traylor M, Benner C, Hsu YH, Richardson TG, et al. Plasma proteomic associations with genetics and health in the UK Biobank. Nature. 2023;622(7982):329–38. 37794186 10.1038/s41586-023-06592-6 PMC10567551

[20] Liu M, Ye Z, Wu Q, Yang S, Zhang Y, Zhou C, et al. Healthy sleep, mental health, genetic susceptibility, and risk of irritable bowel syndrome. J Affect Disord. 2023;331:25–32. 36934852 10.1016/j.jad.2023.03.033

[21] Li X, Zhou T, Ma H, Huang T, Gao X, Manson JE, et al. Healthy sleep patterns and risk of incident arrhythmias. J Am Coll Cardiol. 2021;78(12):1197–207. 34531019 10.1016/j.jacc.2021.07.023 PMC8454031

[22] Fan M, Sun D, Zhou T, Heianza Y, Lv J, Li L, et al. Sleep patterns, genetic susceptibility, and incident cardiovascular disease: a prospective study of 385 292 UK Biobank participants. Eur Heart J. 2020;41(11):1182–9. 31848595 10.1093/eurheartj/ehz849 PMC7071844

[23] Liu Z, Alexander JL, Lin KW, Ahmad T, Pollock KM, Powell N. Infliximab and tofacitinib attenuate neutralizing antibody responses against SARS-CoV-2 ancestral and omicron variants in inflammatory bowel disease patients after 3 doses of COVID-19 vaccine. Gastroenterology. 2023;164(2):300–3. 36270334 10.1053/j.gastro.2022.10.010 PMC9578965

[24] Liu Z, Le K, Zhou X, Alexander JL, Lin S, Bewshea C, et al. Neutralising antibody potency against SARS-CoV-2 wild-type and omicron BA.1 and BA.4/5 variants in patients with inflammatory bowel disease treated with infliximab and vedolizumab after three doses of COVID-19 vaccine (CLARITY IBD): an analysis of a prospective multicentre cohort study. Lancet Gastroenterol Hepatol. 2023;8(2):145–56. 36481043 10.1016/S2468-1253(22)00389-2 PMC9757903

[25] Liu W, Wang T, Zhu M, Jin G. Healthy diet, polygenic risk score, and upper gastrointestinal cancer risk: a prospective study from UK Biobank. Nutrients. 2023. 10.3390/nu15061344. 36986074 10.3390/nu15061344 PMC10054787

[26] Choi SW, Mak TSH, O’Reilly PF. Tutorial: a guide to performing polygenic risk score analyses. Nat Protoc. 2020;15:2759–72. 10.1038/s41596-020-0353-1. 10.1038/s41596-020-0353-1 PMC7612115 32709988

[27] Haonon O, Liu Z, Dangtakot R, Intuyod K, Pinlaor P, Puapairoj A, et al. *Opisthorchis viverrini* infection induces metabolic and fecal microbial disturbances in association with liver and kidney pathologies in hamsters. J Proteome Res. 2021;20(8):3940–51. 34270897 10.1021/acs.jproteome.1c00246

[28] Hu C, Iwasaki M, Liu Z, Wang BC, Li XM, Lin H, et al. Lung but not brain cancer cell malignancy inhibited by commonly used anesthetic propofol during surgery: implication of reducing cancer recurrence risk. J Adv Res. 2021;31:1–12. 34194828 10.1016/j.jare.2020.12.007 PMC8240101

[29] Liu Z, Xia B, Saric J, Utzinger J, Holmes E, Keiser J, et al. Effects of vancomycin and ciprofloxacin on the NMRI mouse metabolism. J Proteome Res. 2018;17(10):3565–73. 30183313 10.1021/acs.jproteome.8b00583

[30] Julkunen H, Cichońska A, Tiainen M, Koskela H, Nybo K, Mäkelä V, et al. Atlas of plasma NMR biomarkers for health and disease in 118,461 individuals from the UK Biobank. Nat Commun. 2023;14(1):604. 36737450 10.1038/s41467-023-36231-7 PMC9898515

[31] Langfelder P, Horvath S. WGCNA: an R package for weighted correlation network analysis. BMC Bioinformatics. 2008;9:559. 19114008 10.1186/1471-2105-9-559 PMC2631488

[32] Friedman J, Hastie T, Tibshirani R. Regularization paths for generalized linear models via coordinate descent. J Stat Softw. 2010;33(1):1–22. 20808728 PMC2929880

[33] Liu Z, Alexander JL, Le KX, Zhou X, Ibraheim H, Anandabaskaran S, et al. Neutralising antibody responses against SARS-CoV-2 Omicron BA.4/5 and wild-type virus in patients with inflammatory bowel disease following three doses of COVID-19 vaccine (VIP): a prospective, multicentre, cohort study. Eclin Med. 2023. 10.1016/j.eclinm.2023.102249. 10.1016/j.eclinm.2023.102249 PMC10570718 37842172

[34] Liu Z, Alexander JL, Eng KY, Ibraheim H, Anandabaskaran S, Saifuddin A, et al. Antibody responses to influenza vaccination are diminished in patients with inflammatory bowel disease on infliximab or tofacitinib. J Crohns Colitis. 2024;18(4):560–9. 37941436 10.1093/ecco-jcc/jjad182 PMC11037107

[35] Johansson E, Olsson T, Strid P, Kockum I, Alfredsson L, Hedström AK. Adolescent sleep patterns, genetic predisposition, and risk of multiple sclerosis. Sleep. 2024. 10.1093/sleep/zsae156. 38975699 10.1093/sleep/zsae156 PMC11467049

[36] Ni J, Zhou Q, Meng SY, Zhou TD, Tian T, Pan HF. Sleep patterns, physical activity, genetic susceptibility, and incident rheumatoid arthritis: a prospective cohort study. BMC Med. 2024;22(1):390. 39272142 10.1186/s12916-024-03615-5 PMC11401439

[37] Sang N, Gao RC, Zhang MY, Wu ZZ, Wu ZG, Wu GC. Causal relationship between sleep traits and risk of systemic lupus erythematosus: a two-sample mendelian randomization study. Front Immunol. 2022;13:918749. 35784289 10.3389/fimmu.2022.918749 PMC9248809

[38] Simon SL, Snell-Bergeon JK, Schäfer M, Barker AJ, Browne LP, Truong U, et al. Sleep duration and association with cardiometabolic health in adolescents and adults with type 1 diabetes: results from the BCQR-T1D study. Diabetes Obes Metab. 2024;26(7):2662–72. 38584515 10.1111/dom.15582 PMC11150084

[39] Yuan S, Sun Y, Tan X, Geng J, Sun J, Chen X, et al. Sleep duration and daytime napping in relation to incident inflammatory bowel disease: a prospective cohort study. Aliment Pharmacol Ther. 2023;57(5):475–85. 36352835 10.1111/apt.17285

[40] Ananthakrishnan AN, Khalili H, Konijeti GG, Higuchi LM, de Silva P, Fuchs CS, et al. Sleep duration affects risk for ulcerative colitis: a prospective cohort study. Clin Gastroenterol Hepatol. 2014;12(11):1879–86. 24780288 10.1016/j.cgh.2014.04.021 PMC4209312

[41] Roth T, Dauvilliers Y, Mignot E, Montplaisir J, Paul J, Swick T, et al. Disrupted nighttime sleep in narcolepsy. J Clin Sleep Med. 2013;9(9):955–65. 23997709 10.5664/jcsm.3004 PMC3746724

[42] Irwin MR, Wang M, Ribeiro D, Cho HJ, Olmstead R, Breen EC, et al. Sleep loss activates cellular inflammatory signaling. Biol Psychiatry. 2008;64(6):538–40. 18561896 10.1016/j.biopsych.2008.05.004 PMC2547406

[43] Irwin MR, Witarama T, Caudill M, Olmstead R, Breen EC. Sleep loss activates cellular inflammation and signal transducer and activator of transcription (STAT) family proteins in humans. Brain Behav Immun. 2015;47:86–92. 25451613 10.1016/j.bbi.2014.09.017 PMC4401620

[44] Sang D, Lin K, Yang Y, Ran G, Li B, Chen C, et al. Prolonged sleep deprivation induces a cytokine-storm-like syndrome in mammals. Cell. 2023;186(25):5500-16.e21. 38016470 10.1016/j.cell.2023.10.025

[45] Vestergaard MV, Allin KH, Poulsen GJ, Lee JC, Jess T. Characterizing the pre-clinical phase of inflammatory bowel disease. Cell Rep Med. 2023;4(11):101263. 37939713 10.1016/j.xcrm.2023.101263 PMC10694632

[46] Liu D, Saikam V, Skrada KA, Merlin D, Iyer SS. Inflammatory bowel disease biomarkers. Med Res Rev. 2022;42(5):1856–87. 35603998 10.1002/med.21893 PMC10321231

[47] Fujino S, Andoh A, Bamba S, Ogawa A, Hata K, Araki Y, et al. Increased expression of interleukin 17 in inflammatory bowel disease. Gut. 2003;52(1):65–70. 12477762 10.1136/gut.52.1.65 PMC1773503

[48] Chen G, Goeddel DV. TNF-R1 signaling: a beautiful pathway. Science. 2002;296(5573):1634–5. 12040173 10.1126/science.1071924

[49] Cabal-Hierro L, Lazo PS. Signal transduction by tumor necrosis factor receptors. Cell Signal. 2012;24(6):1297–305. 22374304 10.1016/j.cellsig.2012.02.006

[50] Zheng X, Zhu Y, Zhao Z, Chu Y, Yang W. The role of amino acid metabolism in inflammatory bowel disease and other inflammatory diseases. Front Immunol. 2023;14:1284133. 37936710 10.3389/fimmu.2023.1284133 PMC10626463

[51] Tang Y, Yu Y, Li R, Tao Z, Zhang L, Wang X, et al. Phenylalanine promotes alveolar macrophage pyroptosis via the activation of CaSR in ARDS. Front Immunol. 2023;14:1114129. 37377971 10.3389/fimmu.2023.1114129 PMC10291621

[52] Parada Venegas D, De la Fuente MK, Landskron G, González MJ, Quera R, Dijkstra G, et al. Short chain fatty acids (SCFAs)-mediated gut epithelial and immune regulation and its relevance for inflammatory bowel diseases. Front Immunol. 2019;10:277. 30915065 10.3389/fimmu.2019.00277 PMC6421268

[53] Meade S, Liu Chen Kiow J, Massaro C, Kaur G, Squirell E, Bressler B, et al. Gut microbiome-associated predictors as biomarkers of response to advanced therapies in inflammatory bowel disease: a systematic review. Gut Microbes. 2023;15(2):2287073. 38044504 10.1080/19490976.2023.2287073 PMC10730146

[54] Johnston AM, Niznikiewicz MM, Gerashchenko D, Strecker RE, Basheer R, Zielinski MR. 0031 Nlrp3 inflammasome mediates Il-18 and Il-18 receptor responses to sleep loss. Sleep. 2018;41(suppl_1):A13-A.

[55] Nowarski R, Jackson R, Gagliani N, de Zoete MR, Palm NW, Bailis W, et al. Epithelial IL-18 equilibrium controls barrier function in colitis. Cell. 2015;163(6):1444–56. 26638073 10.1016/j.cell.2015.10.072 PMC4943028

[56] Cambier S, Gouwy M, Proost P. The chemokines CXCL8 and CXCL12: molecular and functional properties, role in disease and efforts towards pharmacological intervention. Cell Mol Immunol. 2023;20(3):217–51. 36725964 10.1038/s41423-023-00974-6 PMC9890491

[57] Vandendriessche S, Cambier S, Proost P, Marques PE. Complement receptors and their role in leukocyte recruitment and phagocytosis. Front Cell Dev Biol. 2021;9:624025. 33644062 10.3389/fcell.2021.624025 PMC7905230

[58] Neurath MF. Targeting immune cell circuits and trafficking in inflammatory bowel disease. Nat Immunol. 2019;20(8):970–9. 31235952 10.1038/s41590-019-0415-0

[59] Viladomiu M, Khounlotham M, Dogan B, Lima SF, Elsaadi A, Cardakli E, et al. Agr2-associated ER stress promotes adherent-invasive *E. coli* dysbiosis and triggers CD103(+) dendritic cell IL-23-dependent ileocolitis. Cell Rep. 2022;41(7):111637. 36384110 10.1016/j.celrep.2022.111637 PMC9805753

[60] Chang HC, Gau SY. Letter: sleep duration and the incidence of inflammatory bowel disease. Aliment Pharmacol Ther. 2023;57(5):595–6. 36786468 10.1111/apt.17338

