# Supplementary material for: Large-scale plasma proteomics reveals bidirectional associations between sleep patterns and inflammatory bowel disease: a prospective cohort study
Source: BMC Med. 2025 Dec 5;24:18. doi: 10.1186/s12916-025-04539-4 (PMC12797352; doi:10.1186/s12916-025-04539-4)
Supplement: Supplementary file 1 — Additional file 1. Fig. S1. Flowchart outlining the inclusion process for UK Biobank participants in this study. Fig. S2. Identification of co-expression modules using WGCNA. (A) The dendrogram illustrates protein-level dissimilarity and identifies seven co-expression modules. (B) A heatmap depicts eigengene adjacency among the modules, highlighting their inter-module relationships. (C–D) The soft-thresholding power was set to 3, determined by evaluating the scale-free topology index (C) and mean connectivity (D). Fig. S3. LASSO–Cox regression coefficient selection and variable screening. (A) The lower axis denotes the λ values, and the upper axis indicates the corresponding number of variables retained in the LASSO–Cox model. The λ with the minimum cross-validated error is selected. (B) Ten-fold cross-validation is used to determine the optimal tuning parameter in the LASSO–Cox regression model. (C) The bar plot presents the regression coefficients of the 21 proteins retained at the selected λ. (D) The histogram illustrates the distribution of protein scores, with a threshold defined at zero. Fig. S4. Stratified analysis for the association between sleep and IBD. Stratified analysis was performed according to subgroups of demographics and lifestyle behaviors. The logistic model for OR estimation and the Cox model for HR estimation were adjusted by the same confounders as the main analyses. P.int indicated the significance of differences across subgroups. Fig. S5. Performance assessment of protein score-based predictive models. (A) Receiver operating characteristic curves illustrated the prediction accuracy of the protein score employing 16 proteins in the train and test cohorts. AUC is calculated for different follow-up times separately. (B) Cumulative risk curves compared the incident IBD risk between high protein score and low protein score groups in the training and test cohorts, respectively. HR was calculated by the univariable Cox model. Fig. S6. Receiver ope [file 12916_2025_4539_MOESM1_ESM.docx]

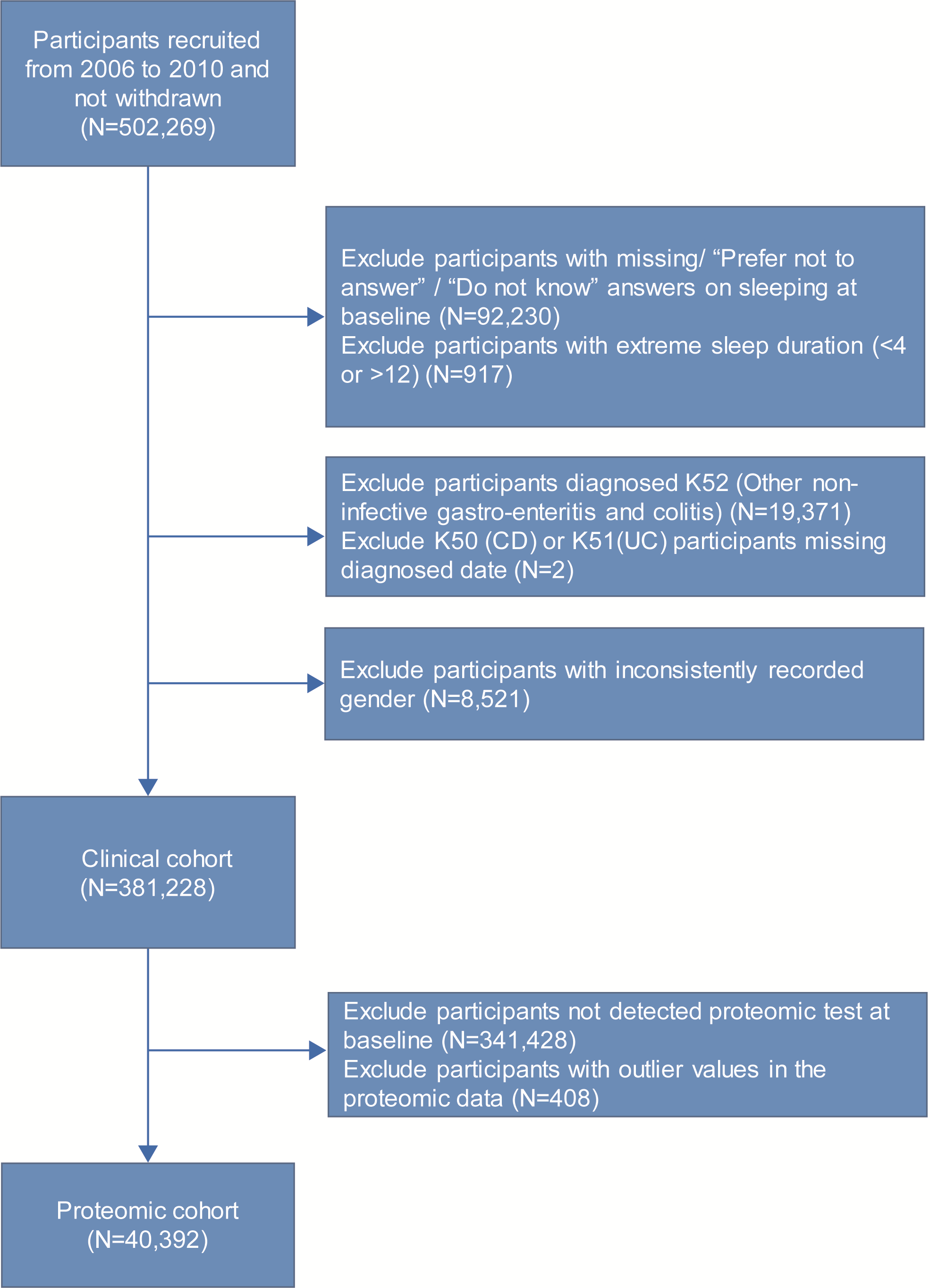


**Fig. S1. Flowchart outlining the inclusion process for UK Biobank participants in this study.**


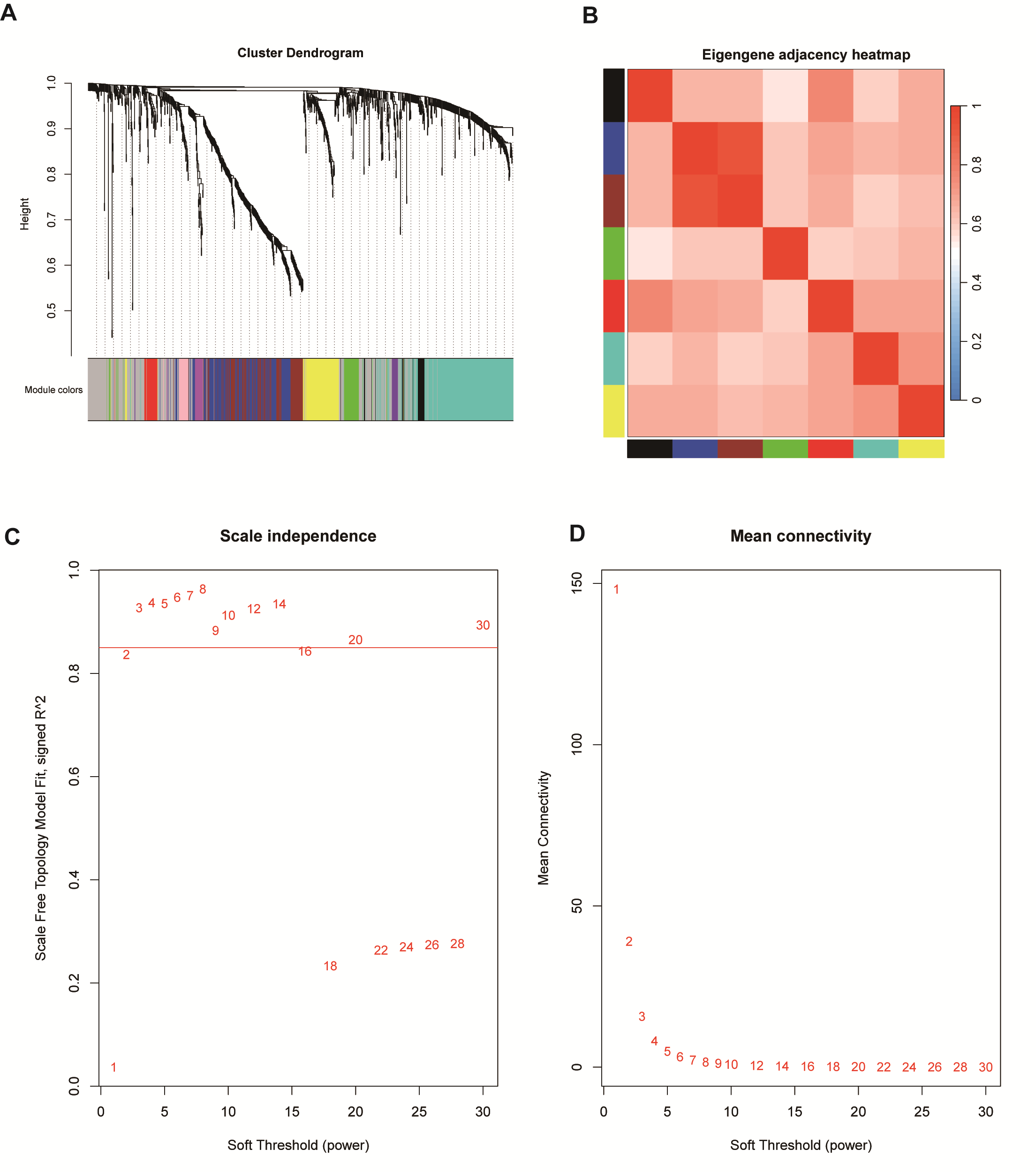


**Fig. S2. Identification of co-expression modules using WGCNA.**

(A) The dendrogram illustrates protein-level dissimilarity and identifies seven co-expression modules.

(B) A heatmap depicts eigengene adjacency among the modules, highlighting their inter-module relationships.

(C–D) The soft-thresholding power was set to 3, determined by evaluating the scale-free topology index (C) and mean connectivity (D).


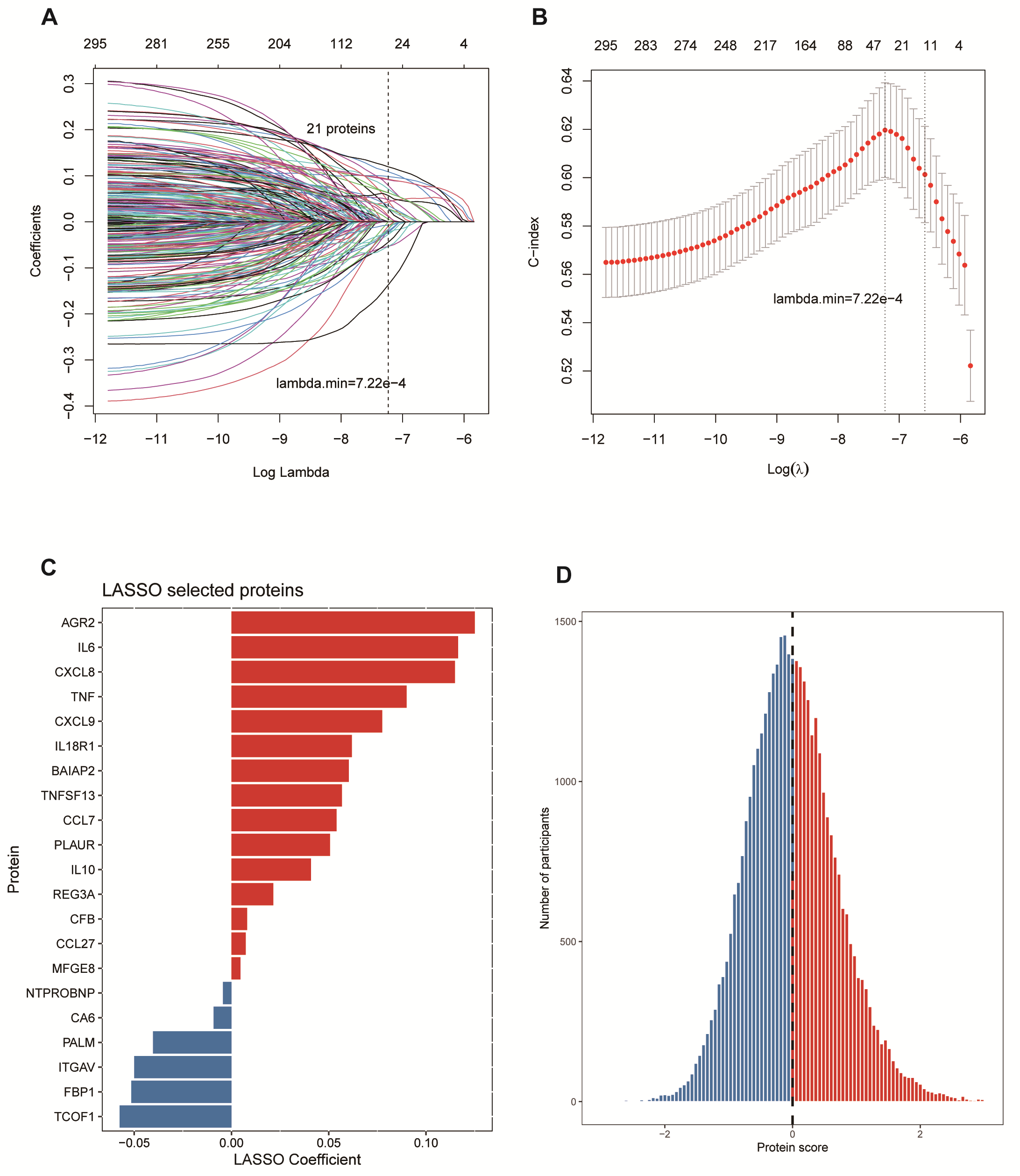


**Fig. S3. LASSO–Cox regression coefficient selection and variable screening.**

(A) The lower axis denotes the λ values, and the upper axis indicates the corresponding number of variables retained in the LASSO–Cox model. The λ with the minimum cross-validated error is selected.

(B) Ten-fold cross-validation is used to determine the optimal tuning parameter in the LASSO–Cox regression model.

(C) The bar plot presents the regression coefficients of the 21 proteins retained at the selected λ.

(D) The histogram illustrates the distribution of protein scores, with a threshold defined at zero.

**
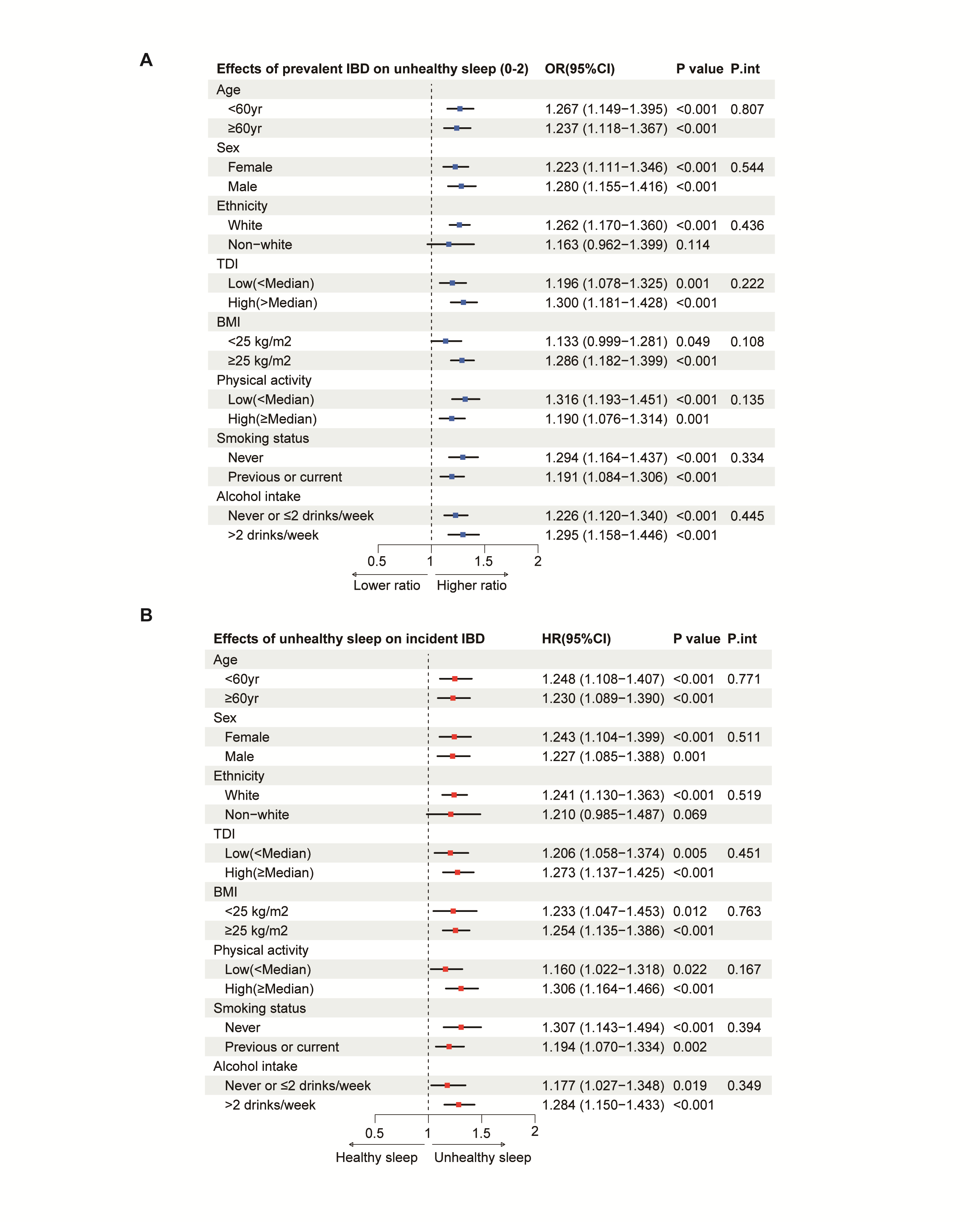
**

**Fig. S4. Stratified analysis for the association between sleep and IBD.**

Stratified analysis was performed according to subgroups of demographics and lifestyle behaviors. The logistic model for OR estimation and the Cox model for HR estimation were adjusted by the same confounders as the main analyses. P.int indicated the significance of differences across subgroups.


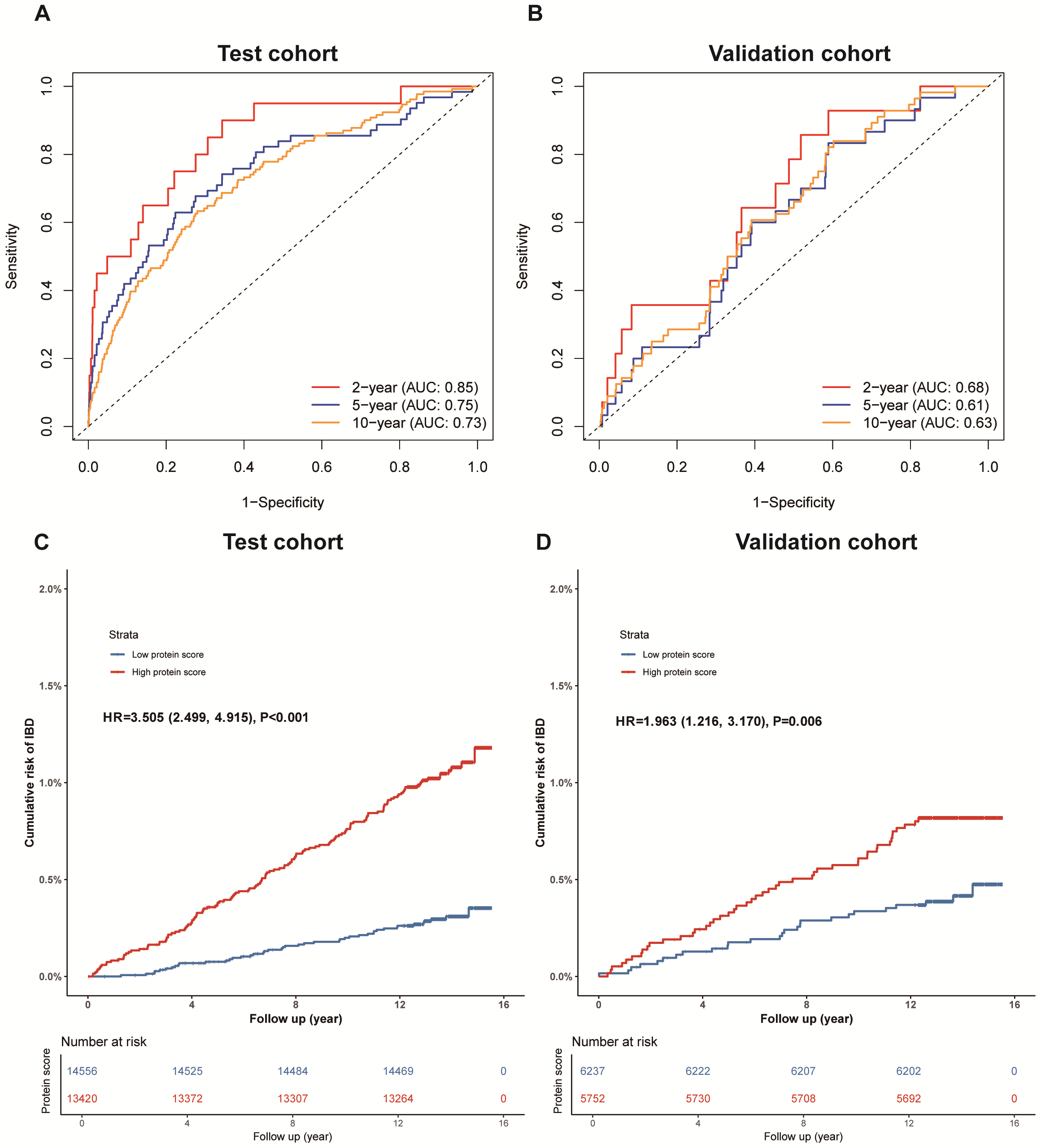


**Fig. S5. Performance assessment of protein score-based predictive models.**

(A) Receiver operating characteristic curves illustrated the prediction accuracy of the protein score employing 16 proteins in the train and test cohorts. AUC is calculated for different follow-up times separately. (B) Cumulative risk curves compared the incident IBD risk between high protein score and low protein score groups in the training and test cohorts, respectively. HR was calculated by the univariable Cox model.


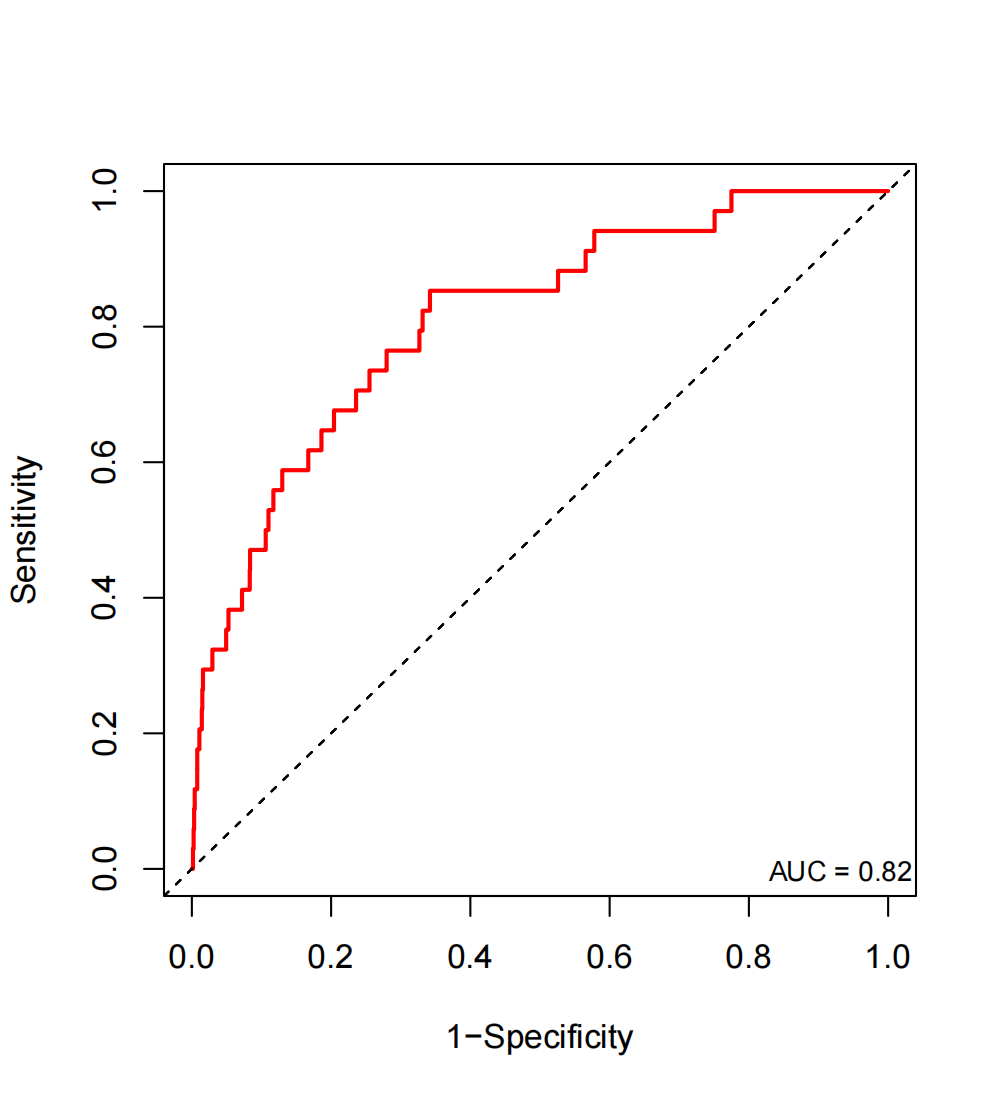


**Fig. S6**. Receiver operating characteristic curve of the 2-year IBD prediction model incorporating combined sleep scores (with demographic and lifestyle covariates), proteomic score, and polygenic risk score (PRS). Proteomic data included all measured proteins, including those with values below the detection limit (except those of poor assay quality).
